# Standard Binding Free Energy and Membrane Desorption Mechanism for a Phospholipase C

**DOI:** 10.1021/acs.jcim.1c01543

**Published:** 2022-03-28

**Authors:** Emmanuel E. Moutoussamy, Hanif M. Khan, Mary F. Roberts, Anne Gershenson, Christophe Chipot, Nathalie Reuter

**Affiliations:** † Department of Biological Sciences, University of Bergen, N-5020 Bergen, Norway; ‡ Computational Biology Unit, Department of Informatics, University of Bergen, N-5020 Bergen, Norway; § Department of Chemistry, Boston College, Chestnut Hill, Massachusetts 02467, United States; ∥ Department of Biochemistry and Molecular Biology, University of Massachusetts Amherst, Amherst, Massachusetts 01003, United States; ⊥ Laboratoire International Associé Centre National de la Recherche Scientifique et University of Illinois at Urbana−Champaign, Unité Mixte de Recherche n 7019, Université de Lorraine, BP 70239, 54506 Vandœuvre-lès-Nancy cedex, France; # Department of Physics, University of Illinois, Urbana, Illinois 61801, United States; ∇ Department of Chemistry, University of Bergen, N-5020 Bergen, Norway

## Abstract

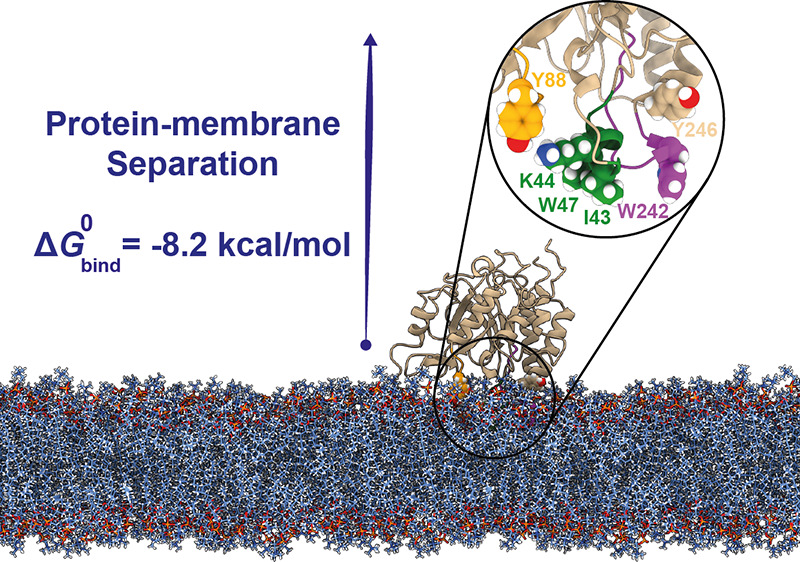

Peripheral membrane proteins (PMPs) bind temporarily to cellular membranes and play important roles in signaling, lipid metabolism, and membrane trafficking. Obtaining accurate membrane-PMP affinities using experimental techniques is more challenging than for protein–ligand affinities in an aqueous solution. At the theoretical level, calculation of the standard protein–membrane binding free energy using molecular dynamics simulations remains a daunting challenge owing to the size of the biological objects at play, the slow lipid diffusion, and the large variation in configurational entropy that accompanies the binding process. To overcome these challenges, we used a computational framework relying on a series of potential-of-mean-force (PMF) calculations including a set of geometrical restraints on collective variables. This methodology allowed us to determine the standard binding free energy of a PMP to a phospholipid bilayer using an all-atom force field. *Bacillus thuringiensis* phosphatidylinositol-specific phospholipase C (*Bt*PI-PLC) was chosen due to its importance as a virulence factor and owing to the host of experimental affinity data available. We computed a standard binding free energy of −8.2 ± 1.4 kcal/mol in reasonable agreement with the reported experimental values (−6.6 ± 0.2 kcal/mol). In light of the 2.3-μs separation PMF calculation, we investigated the mechanism whereby *Bt*PI-PLC disengages from interactions with the lipid bilayer during separation. We describe how a short amphipathic helix engages in transitory interactions to ease the passage of its hydrophobes through the interfacial region upon desorption from the bilayer.

## 1 Introduction

Peripheral membrane proteins (PMPs) are soluble proteins, which temporarily bind to cellular membranes with exquisite resolution in time and space. They play important roles in a host of key physiological processes, including signaling, lipid metabolism, membrane trafficking, and pathogen toxicity.^1−7^ PMPs bind to cellular membranes by shallowly inserting loops and/or an amphipathic helix in the chemically complex environment of the membrane interface, and many PMPs recognize particular lipid compositions. Information about the thermodynamics of PMP-membrane association is, however, needed to improve our understanding of the mechanisms at play in lipid recognition. There is an array of experimental techniques for measuring membrane-PMP affinities,^8^ but obtaining accurate data is more challenging than for protein–ligand affinities in an aqueous solution. Experimental difficulties in quantifying membrane-PMP affinities include heterogeneity in vesicle sizes, the potential for protein aggregation in solution or at the surface of a vesicle, and often vesicle aggregation and fusion occurring over time.

Computational determination of the standard free energy of binding of two molecular partners based on molecular dynamics (MD) simulations can be quite accurate for protein–ligand^9^ and protein–protein association in an aqueous environment,^10^ assuming the use of a suitable methodology, an accurate force field, and adequate sampling. In comparison to solution protein–ligand association events, PMP-membrane association brings additional challenges related to the size of the biological objects at play and the large change in configurational entropy accompanying the binding process. Another challenge pertains to the lateral diffusion of lipids in the bilayer, typically on the order of 10^–7^ to 10^–8^ cm^2^ s^–1^,^11,12^ which is slow compared to tractable simulation times for such systems. We have shown that calculation of relative binding free energies using free-energy perturbation (FEP) can reproduce the experimental difference in dissociation constants measured when interfacial aromatic residues were substituted by alanine in three PMPs,^13^ yet and to the best of our knowledge, determination of absolute membrane binding free energies for PMPs has only been achieved using implicit membrane models^14^ or coarse-grained force fields. Using MD simulations with the MARTINI 2.1 CG force field,^15,16^ Naughton et al. have evaluated the free energy of binding of the GRP1 pleckstrin homology (PH) domain to PI(3,4,5)P_3_ or PI(4,5)P_2_ embedded in lipid bilayers.^17^ The free energy was calculated from the potential of mean force (PMF) along the protein–membrane separation using umbrella sampling (US).^18^ The same computational strategy was later applied to phosphatidylinositol phosphates binding to 12 different PH domains.^19^ Using US and an implicit solvent/membrane model, Zhang et al. estimated the membrane binding free energy of peptides of various lengths, including the flexible 25-amino acid long MARCKS-ED peptide.^14^ Compared to atomistic simulations, both implicit and CG membrane models accelerate sampling at the price of sacrificing the fine detail of the interfacial PMP-lipid interactions, which have been shown to be crucial for lipid recognition.^13^

Here, we performed all-atom MD simulations to estimate the standard binding free energy of *Bacillus thuringiensis* phosphatidylinositol-specific phospholipase C (*Bt*PI-PLC) with a dimyristoylphosphatidylcholine (DMPC) lipid bilayer. *Bt*PI-PLC, a 296-amino acid long enzyme secreted by the Gram-positive bacterium *B. thuringiensis*, reduces host innate immunity^20^ by catalyzing the cleavage of glycophosphatidylinositol (GPI)-anchored proteins at the surface of the cell, thereby contributing to bacterial virulence.^21−23^*Bt*PI-PLC binds preferentially to vesicles rich in PC lipids, albeit also containing a small fraction (ca. 10–20% mol) of anionic lipids.^24,25^ The availability of detailed experimental data for *Bt*PI-PLC binding to lipid vesicles makes it a convenient choice to evaluate our computational approach beyond simple models.^8,13,25−28^ Apparent dissociation constants (*K*_D_) for *Bt*PI-PLC on small unilamellar POPC vesicles have been determined experimentally for an array of membrane compositions and protein variants.^25−29^ Because of the heterogeneity of small unilamellar vesicle sizes these have been reported in terms of total phospholipid concentration of the vesicle rather than phospholipid in the outer monolayer. The average of the reported *K*_D_ values for WT *Bt*PI-PLC on POPC SUVs is 24 ± 6 μM (Table SI.1). Correcting for only the phospholipid in the outer monolayer yields an average *K*_D_ for accessible POPC between 18.0 ± 4.5 and 15.6 ± 3.9 μM and Δ*G*^0^_bind_ of −6.6 ± 0.2 kcal/mol (for details of the calculation see Table S1).

**Figure 1:**
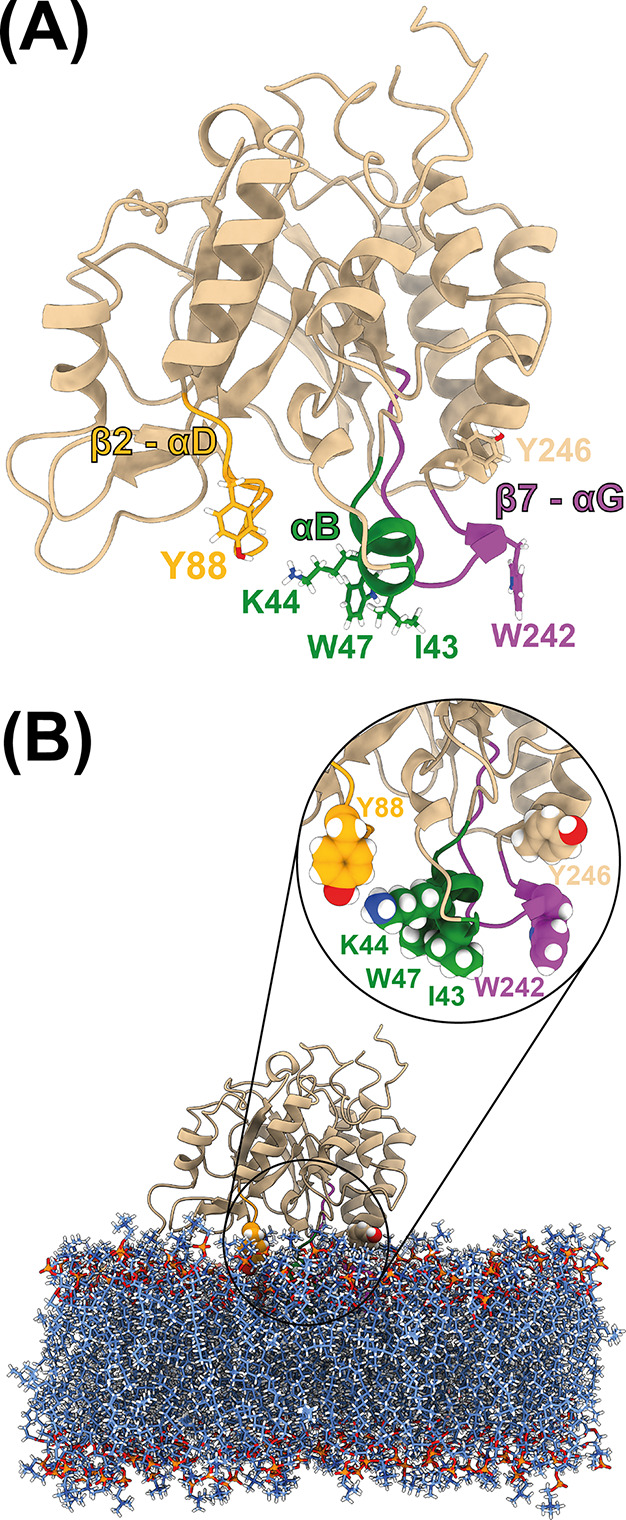
Structure of *Bt*PI-PLC (A) and membrane-bound *Bt*PI-PLC (B). The loops involved in the IBS are shown in green (helix B), purple (rim loop, β7-αG), and orange (β2-αD), with sticks (A) or spheres (B) for residues Ile43, Lys44, Trp47, Tyr88, Trp242, and Tyr246. The DMPC lipids are shown with spheres colored by atom types (blue for C, dark blue for N, red for O, orange for P, and white for H).

The *Bt*PI-PLC interfacial binding site (IBS) consists of a small amphipathic helix (helix B, green in Figure 1) and two neighboring loops, including the rim loop connecting β-strand 7 and α-helix G (colored purple in Figure 1). The two loops are rich in surface-exposed tyrosine amino acids,^26,29,30^ which have been shown to be essential for PC lipid recognition through the determination of *K*_D_ for tyrosine-to-alanine mutants^13,25,26,28^ and by engineering PC recognition into *Staphylococcus aureus* PI-PLC via the strategic introduction of two tyrosine mutations.^27,31^ In MD simulations of the membrane-bound *Bt*PI-PLC, using the all-atom CHARMM36m force field, we observed that tyrosine residues engage in cation−π interactions with choline headgroups and that Tyr88 and Tyr246 form the most stable interactions with choline headgroups. The Tyr-choline interactions have been confirmed experimentally,^27,31,32^ and our estimates of the individual tyrosine contributions to the overall binding free energy are as much as 2.5 kcal/mol.^13,26^ In addition to interactions of tyrosine amino acids with the membrane, the short helix B (7–8 amino acids) contains an isoleucine (I43) and a tryptophan (W47) that insert below the phosphate groups.^26,33,34^ Lysine 44 (K44) in helix B is also important for *Bt*PI-PLC membrane binding. It contributes to the latter through its ammonium group, which forms a salt bridge with the phosphate group, but also via contacts between its aliphatic chain and the lipid acyl chains. However, little is known about the actual binding mechanism of *Bt*PI-PLC and how equilibrium interactions are established at the membrane interface.

In order to calculate the membrane absolute binding free energy, we used the geometrical route introduced by Gumbart et al. in 2013.^35^ This approach^35−37^ consists of introducing geometrical restraints to reduce the large change in configurational entropy that accompanies the association of a protein to the surface of lipid bilayers and the precise evaluation of the free-energy contribution arising from these geometrical restraints. In practice, the host–guest binding event is decomposed into several subprocesses, each of them describing a degree of freedom of the guest with respect to its host. Geometrical restraints are progressively added to these degrees of freedom, and their contributions are evaluated by means of independent PMF calculations. Finally, the absolute binding free energy is recovered by integration of these PMFs. In our case, the PMP (*Bt*PI-PLC) is considered as the guest of a host lipid bilayer (Figure 1). This framework has been successfully employed to evaluate the standard binding free energy underlying protein–ligand,^35^ protein–protein,^10^ and DNA-ligand^38^ association. In this work, we demonstrate the applicability of the approach to protein–membrane binding and use the generated MD trajectories to shed new light on the mechanism of reversible desorption of hydrophobic and aromatic residues from the aqueous interface, as well as on the role of the interfacial K44 residue in this process.

## 2 Methods

### 2.1 Simulations Setup

In order to calculate the absolute membrane binding free energy of *Bt*PI-PLC to a DMPC bilayer, we used the computational framework introduced by Gumbart et al. in 2013.^35^ Construction of three different computational assays was necessary to achieve this goal. They are labeled system 1 to system 3 in Table 1, as well as in the rest of this contribution.

**Table 1:** Composition and Dimensions of the Simulated Systems

system name		composition	number of atoms	box dimensions
1	*Bt*PI-PLC with bilayer	*Bt*PI-PLC	96328	89 Å × 89 Å × 118 Å
		256 DMPC		
		20453 TIP3P		
		7 Na^+^		
2	*Bt*PI-PLC with bilayer	*Bt*PI-PLC	138717	89 Å × 89 Å × 170 Å
		256 DMPC		
		34586 TIP3P		
		7 Na^+^		
3	*Bt*PI-PLC in water	*Bt*PI-PLC	78243	90 Å × 90 Å × 95 Å
		24494 TIP3P		
		7 Na^+^		
4	Y247S/Y251S	*Bt*PI-PLC	46990	80 Å × 80 Å × 80 Å
	*Bt*PI-PLC in water	14083 TIP3P		
		7 Na^+^		

The starting model for wild-type (WT) *Bt*PI-PLC was built from the X-ray crystallographic structures of two *Bt*PI-PLC mutants: Y247S/Y251S (PDB ID: 3EA1(29)) and W47A/W242A (PDB ID: 2OR2(39)), as described in ref (26). The p*K*_a_ values of ionizable side chains were predicted using PROPKA3,^40,41^ and none indicated a deviation from the standard protonation states of individual amino acids at pH 7. The resulting WT model was immersed in a cubic box of 24,494 water molecules and neutralized with seven sodium ions to build system 3 (Table 1). The same WT model was used to build systems 1 and 2, wherein *Bt*PI-PLC is bound to a 256-DMPC lipid bilayer.

In addition, we performed a simulation of a *Bt*PI-PLC variant in water (Y247S/Y251S, PDB ID: 3EA1).^29^ The X-ray structure was hydrated in a cube of 14,083 water molecules including crystallographic water and neutralized with seven sodium ions (system 4). This simulation was performed in order to obtain additional structural information on side chain orientation and solvation at the membrane binding site.

#### 2.1.1 Protocol for Protein Adsorption on Lipid Bilayers (Systems 1 and 2)

System 1 was prepared for the study reported in ref (13). Briefly, we ran a simulation using the Highly Mobile Membrane Mimetic (HMMM) model^42^ to accelerate protein adsorption onto the DMPC bilayer. The protein was placed above a HMMM bilayer such that the shortest protein–membrane distance was 5 Å and oriented with the interfacial binding site facing the membrane. The system was built using the HMMM builder^43^ of the CHARMM-GUI,^44^ and a 200-ns simulation was performed at 310 K. During this simulation, we applied the recommended harmonic restraints to the lipid bilayer.^42^ The last configuration of the simulation was converted to a system with a full-tail lipid bilayer and subjected to a geometry optimization with conjugate gradients (CGs) for 20,000 steps followed by 50 ns of equilibration in the isobaric–isothermal ensemble at 310 K and 1 atm. System 2 was prepared by adding an additional layer of solvent to system 1. Both systems 1 and 2 were then subjected to a geometry optimization with CGs for 10,000 steps, followed by a 50-ns MD simulation for 50 ns in the isobaric–isothermal ensemble. The protonation state of titratable residues was considered, but none of the lysines or arginines in the IBS were inserted deep enough to warrant a change in the protonation state prior to or during the simulations.^45−47^ Likewise there were no aspartic and glutamic acids located at the membrane interface;^45,48^ therefore, we do not expect changes in the protonation states for any of these amino acids.

#### 2.1.2 Preparation of Systems for Simulations of *Bt*PI-PLC in Water (Systems 3 and 4)

Systems 3 and 4 were subjected to an energy minimization consisting of 5,000 steps of CG. Both systems were equilibrated for 150 ps in the isobaric–isothermal ensemble with harmonic restraints applied to backbone atoms with a force constant of 1.0 kcal/mol/Å^2^, followed by another 100-ps equilibration devoid of geometric restraints. Finally, a 100-ns simulation was performed for each system. We monitored the backbone distance RMSD with respect to the last structure after the second equilibration step to verify that the protein structure was robust over the time scale of the simulations (Figure S1).

#### 2.1.3 Molecular Dynamics Simulation Parameters

All simulations were performed using the program NAMD2.13,^49^ with the CHARMM36m force field,^50^ including the NBFIX corrections for ions, and the CHARMM-WYF extension for choline-aromatics cation−π interactions.^51,52^ The temperature was set at 310 K and controlled by Langevin dynamics, with a damping coefficient of 1.0 ps^–1^. The pressure control was semi-isotropic. We used the Langevin piston method (target pressure: 1 atm).^53^ The SETTLE algorithm^54^ was used to constrain water molecules to their equilibrium geometry, and RATTLE was applied to constrain all other chemical bonds involving hydrogen atoms.^55^ An integration time step of 2 fs was used to integrate the equations of motion. Short-range electrostatics and van der Waals interactions were truncated smoothly between 10 and 12 Å. The particle-mesh Ewald algorithm was employed to account for long-range electrostatic interactions.^56^

### 2.2 Membrane Binding Free Energy Calculations

#### 2.2.1 Theoretical Background

**Figure 2:**
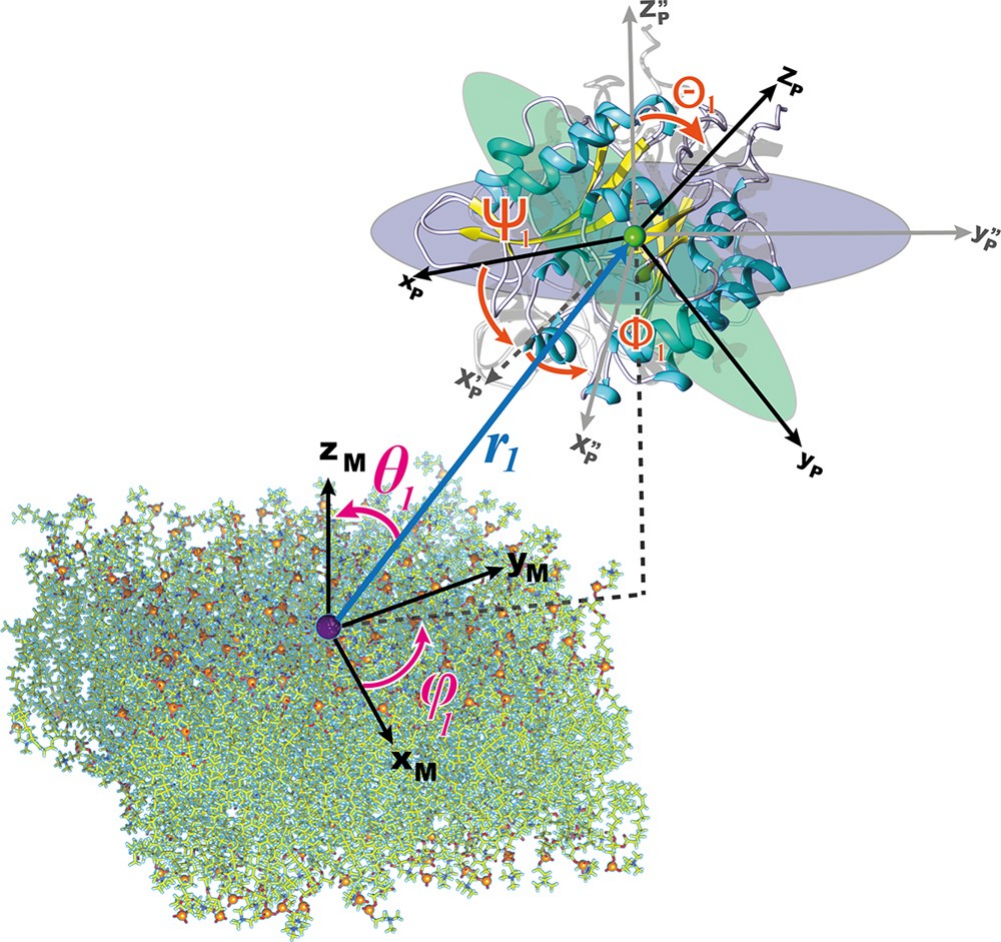
Degrees of freedom considered for the protein–membrane absolute binding free energy calculation. Euler angles, Θ_1_, Φ_1_, and Ψ_1_, and polar and azimuthal angles, *θ*_1_ and *φ*_1_, describe the relative orientation and position of the protein with respect to the bilayer.

As stated earlier, the computational approach used here consists of applying a series of geometrical restraints in order to reduce the degrees of freedom of the protein with respect to the bilayer. The degrees of freedom considered to restrain the protein in the bound form are depicted in Figure 2.

Convergence of the separation PMF calculation was accelerated by means of the geometric restraints enforced on these degrees of freedom. The contribution for adding these restraints in the bound state, or alternatively, removing them in the unbound state, was accounted for in the final calculation of the absolute binding free energy. The full description of the methodology can be found in the article of Gumbart et al., 2013.^35^ From the eight PMF calculations listed in Table 2, the equilibrium constant (*K*_eq_) can be determined using eq 1
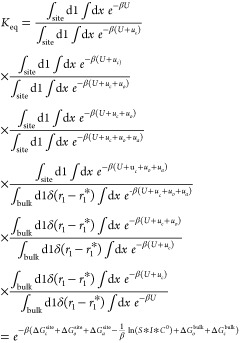
1where *u*_0_ = *u*_Θ_ + *u*_Φ_ + u_Ψ_ (orientational restraining potential), and *u*_*a*_ = *u*_θ_1__ + *u*_φ_1__ (positional restraining potential). *U* is the potential energy, and *u*_*c*_ is the conformational restraining potential. *C*^0^ is the standard state concentration of 1 M , and  (*k*_B_ is the Boltzmann constant, and *T* is the temperature.). 1 denotes the guest in the guest/host complex, which, in this case, corresponds to the protein. *r*_1_ is the position of the protein COM, and *r*_1_^*^ is some location far from the binding site. The subscripts “site” and “bulk” refer to the bound and the unbound states of the protein–membrane complex.

**Table 2:** Eight PMF Calculations Performed to Obtain Δ*G*_bind_^0^^a^

PMF	collective variable	restraints	system used (cf. Table 2)
1	RMSD		1
2	Θ_1_	protein backbone	1
3	Φ_1_	protein backbone and Θ_1_	1
4	Ψ_1_	protein backbone, Θ_1_, and Φ_1_	1
5	*θ*_1_	protein backbone, Θ_1_, Φ_1_, and Ψ_1_	1
6	*φ*_1_	protein backbone, Θ_1_, Φ_1_, Ψ_1_, and *θ*_1_	1
7	*r*_1_	protein backbone, Θ_1_, Φ_1_, Ψ_1_, *θ*_1_, and *φ*_1_	2
8	RMSD		3

a *r*_1_ is the distance between the center of mass (COM) of the protein and that of the upper phosphate plane. The conformational restraints (RMSD) were calculated for the protein backbone.

The fourth terms of eq 1 can be reformulated introducing the terms S* and I*, such as
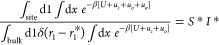
2where S* corresponds to the sphere surface of radius *r*_1_^*^ and is centered on the membrane-protein binding site, accessible to the protein. I* contains the separation PMF
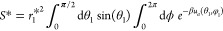
3
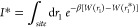
4where *W*(*r*_1_) is the separation PMF. A noteworthy difference with the case of protein–ligand association in an aqueous environment–which is expected to be isotropic–is the anisotropic approach of the protein toward the bilayer; the protein can explore only half of the polar angles. In order to take this difference into account in our calculations, we divided the integration limits of the first polar angle, *θ*_1_, in the S* term by two. The fifth term of eq 1 (Δ*G*_*o*_^bulk^) corresponds to the reorientation of a rigid body (the protein restrained in its native conformation when bound to the membrane) and can, therefore, be evaluated analytically. The other contributions need to be determined using MD simulations (Table 2). *K*_eq_ can then be converted to the binding free energy using
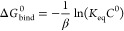
5

#### 2.2.2 Potential of Mean Force (PMF) Calculations

The eight PMF calculations were performed using the extended-Lagrangian version of the Adaptive Biasing Force algorithm (eABF).^57,58^ To sample efficiently the transition coordinate in each PMF calculation, we split the reaction pathway into a suitable number of overlapping windows (stratification strategy^59^). The starting structures for each window were extracted from the overlapping region between adjacent windows. The instantaneous force was collected in bins 1°, 0.05 Å, and 0.1 Å wide for the angular (Θ_1_, Φ_1_, Ψ_1_, *θ*_1_, and *φ*_1_), the RMSD, and the separation (*r*_1_) PMFs, respectively. A force constant of 0.1 kcal/mol·deg^2^ was used for restraining the orientation (Θ_1_, Φ_1_, and Ψ_1_) and the polar and azimuthal angles (*θ*_1_ and *φ*_1_) of the protein with respect to the bilayer. The protein backbone was positionally restrained using a force constant of 15 kcal/mol·Å^2^. The thresholds^60^ for applying the bias were set to 100,000 and 50,000 force samples per bin for, respectively, the separation PMF, on the one hand, and the other PMF calculations, on the other hand.

#### 2.2.3 Statistical Error Calculation

The statistical errors were calculated using the method described in Comer et al., 2015.^61^ The error of the mean force in bin *i* can be estimated with eq 6
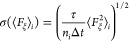
6where ξ is the reaction coordinate, τ is the correlation length of the time series, *n*_*i*_ is the number of force samples in bin *i*, Δ*t* is the time step used for the simulation, and ⟨*F*_ξ_^2^⟩_*i*_ is the variance of the force in bin *i*. We calculated the variance of the force and the correlation length of the time series based on a short simulation, where only one bin was sampled, and no bias was applied. ⟨*F*_ξ_^2^⟩_*i*_ and τ were considered to be constant across the reaction coordinate. The statistical error was then propagated to the full PMF using the Bienaymé formula:^62^
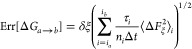
7

### 2.3 Trajectory Analysis

For each of the simulations in water (systems 3 and 4), the distance RMSD for the protein backbone between simulation frames and the structure obtained after the last equilibration step was monitored along the simulations (Figure S1). We evaluated the depth of anchorage mostly by looking at electron density profiles (EDPs) of the protein and various lipid chemical groups. Distance RMSD and EDPs were calculated using VMD.^63^ Analysis of the trajectories was otherwise performed using MDAnalysis.^64,65^ We analyzed two types of trajectories: those from equilibrium simulations (systems 3 and 4) and those generated during the separation PMF (system 2, PMF 7 in Table 2). We identified hydrophobic contacts, hydrogen bonds, and cation−π interactions using the same definitions as in Grauffel et al.^26^ Hydrophobic contacts were considered to exist if two candidate atoms, not covalently bonded, were within 3 Å of each other for a duration of at least 10 ps. Candidate atoms are those belonging to aliphatic groups of amino-acid side chains and lipid chains. Hydrogen bonds were defined by a hydrogen-donor distance below or equal to 2.4 Å and an angle between donor, hydrogen, and acceptor higher than or equal to 130°. Finally, cation−π interactions between the aromatic rings of tyrosine and tryptophan residues were considered to exist when all distances between the aromatic-ring heavy atoms and the choline nitrogen atom were below 7 Å. In addition, these distances should not differ by more than 1.5 Å from each other. The separation PMF trajectory was divided into 31 windows each corresponding to a value of *r*_1_ (15 to 45 Å, increments of 1 Å), and 2000 frames were collected in each window. The calculation of the average number of water molecules around W47 along the separation PMF was done as follows. Water molecules were counted if their oxygen atom was within 5 Å of any atom of W47. The count was done for each value of *r*_1_ from 15 to 45 Å (increments of 1 Å), and averages were calculated. The analyses of systems 3 and 4 were performed over the whole 100-ns production runs. Images from the simulations were generated using UCSF Chimera.^66^

## 3 Results

### 3.1 Standard Binding Free Energy of BtPI-PLC on a DMPC Bilayer

**Figure 3:**
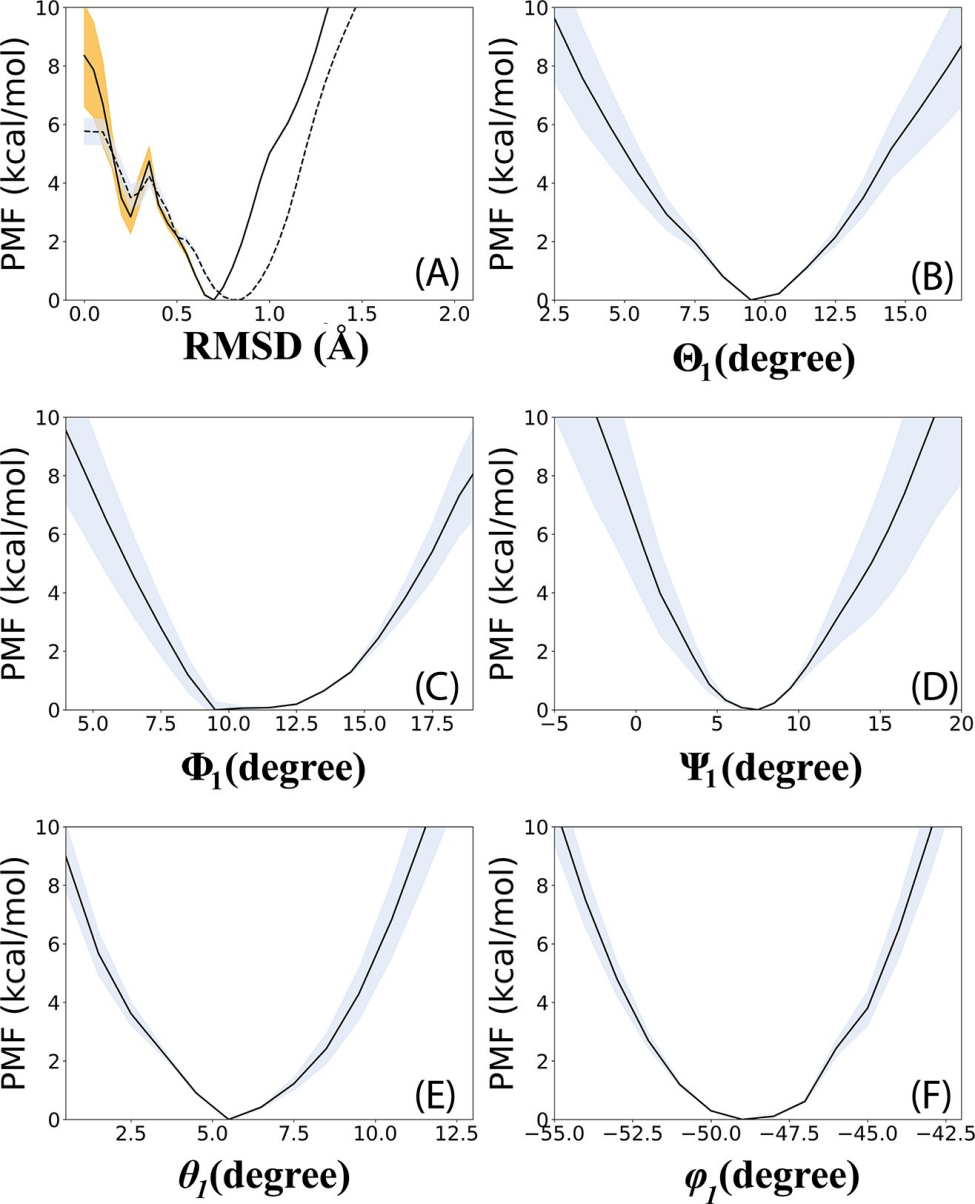
Conformational, positional, and orientational PMFs. (A) RMSD in the membrane-bound state (solid line) and in the bulk (dashed line), (B–D) the three Euler angles (Θ_1_, Φ_1_, and Ψ_1_), and (E and F) the two polar angles (*θ*_1_ and *φ*_1_) of membrane-bound *Bt*PI-PLC. The error bars are indicated by the orange and blue shading for the RMSD and angles, respectively.

**Figure 4:**
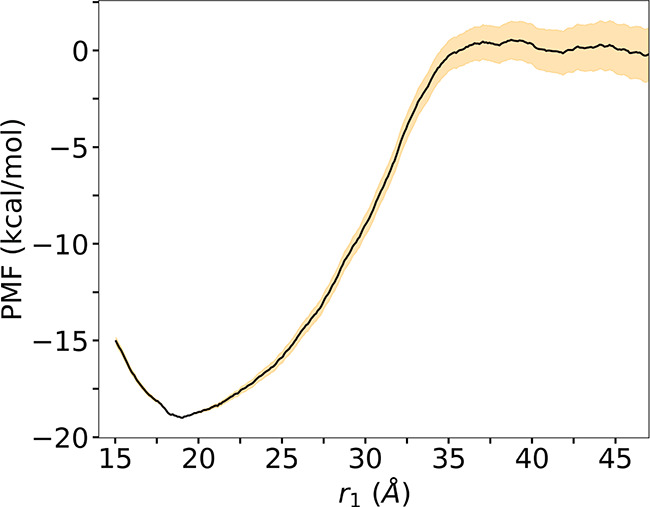
Separation PMF. The standard deviation is indicated in orange. *r*_1_ is the distance between the COM of *Bt*PI-PLC and the COM of the upper phosphate plane of the DMPC bilayer.

The contributions to the binding free energy are reported in Table 3, together with the corresponding statistical errors and simulation times, which taken altogether correspond to an aggregate time of 2.7 μs. The PMFs are shown in Figures 3 and 4.

**Table 3:** Computed and Experimentally Determined Free Energies of Binding^b^

contribution	PMF (kcal/mol)	time (ns)
Δ*G*_*c*_^site^	–3.0 ± 0.3	100
Δ*G*_Θ_1__^site^	0.0 ± 0.0	30
Δ*G*_Φ_1__^site^	–0.1 ± 0.0	30
Δ*G*_Ψ_1__^site^	–0.2 ± 0.2	35
Δ*G*_θ_1__^site^	0.0 ± 0.0	50
Δ*G*_φ_1__^site^	–0.1 + 0.0	50
–(1/β) ln(S*I*C^0^)	–15.9 ± 1.3	2300
Δ*G*_*o*_^bulk^	+7.6	
Δ*G*_*c*_^bulk^	+3.7 ± 0.2	80
Δ*G*_bind comp_^0^	–8.2 ± 1.4	2675
Δ*G*_bind exp_^0^	–6.6 ± 0.2^a^	

a See the SI for explanation of the experimental determination of Δ*G*_bind exp_^0^.

b Each contribution to the computed Δ*G*_bind_^0^ is also provided together with the length of the corresponding simulation.

The contributions of orientational (*G*_*o*_^site^ = Δ*G*_Θ_1__^site^ + Δ*G*_Φ_1__^site^ + Δ*G*_Ψ_1__^site^) and positional (*G*_*a*_^site^ = Δ*G*_θ_1__^site^ + Δ*G*_φ_1__^site^) restraints in the bound state are small: −0.3 and −0.1 kcal/mol, respectively. They are of comparable magnitude to what has been obtained for protein–protein,^10^ protein-peptide,^35^ and protein–DNA^38^ association using the same theoretical framework. The PMFs characterizing the protein conformational change at the surface of the bilayer and in the bulk have comparable shapes (Figure 3A), but they are offset. The corresponding contributions are Δ*G*_*c*_^site^ = −3.0 ± 0.3 kcal/mol and Δ*G*_*c*_^bulk^ = +3.7 ± 0.2 kcal/mol, respectively. As expected, convergence of the separation PMF required the largest amount of computational time, that is, 2.3 μs or 86% of 2.675 μs required for the entire set of simulations. The well depth of the separation profile is ca. 19 kcal/mol (Figure 4), and its related contribution to the free energy is −15.9 kcal/mol (−(1/β) ln(S*I*C^0^), Table 3) since I* contains the separation PMF. The free-energy minimum for *Bt*PI-PLC bound to the DMPC bilayer was obtained for *r*_1_ = 18 Å in agreement with the distance measured in an equilibrium MDs simulation of the complex (Figure S2). *r*_1_ = 18 Å corresponds to a minimum protein-bilayer distance of 1.7 Å (Figure S3). The complete separation of *Bt*PI-PLC from the bilayer occurred at ca. *r*_1_ = 35 Å, where the minimum distance between protein and lipids starts increasing (Figure S3).

The binding free energy was computed following eq 1 in the Methods section and is detailed in the Supporting Information. We used three different values for *r*_1_^*^ (41, 44, and 46 Å) leading to values of −8.2, −8.5, and −8.2 kcal/mol for Δ*G*_bind_^0^, respectively.

### 3.2 Desorption of the Short Amphipathic Helix B

Helix B, a short amphipathic helix (Figure 1, P42 to G48), is critical for the affinity of *Bt*PI-PLC for phospholipid bilayers.^8,33,34^ Earlier MDs simulations showed that helix B is deeply anchored at the interface and that I43, K44, and W47 engage in hydrophobic contacts with the surrounding lipids.^25,26^ We followed the interaction of these three helix B residues with lipids during the separation PMF in order to understand which strategies they use to cross the polar region of the membrane interface.

**Figure 5:**
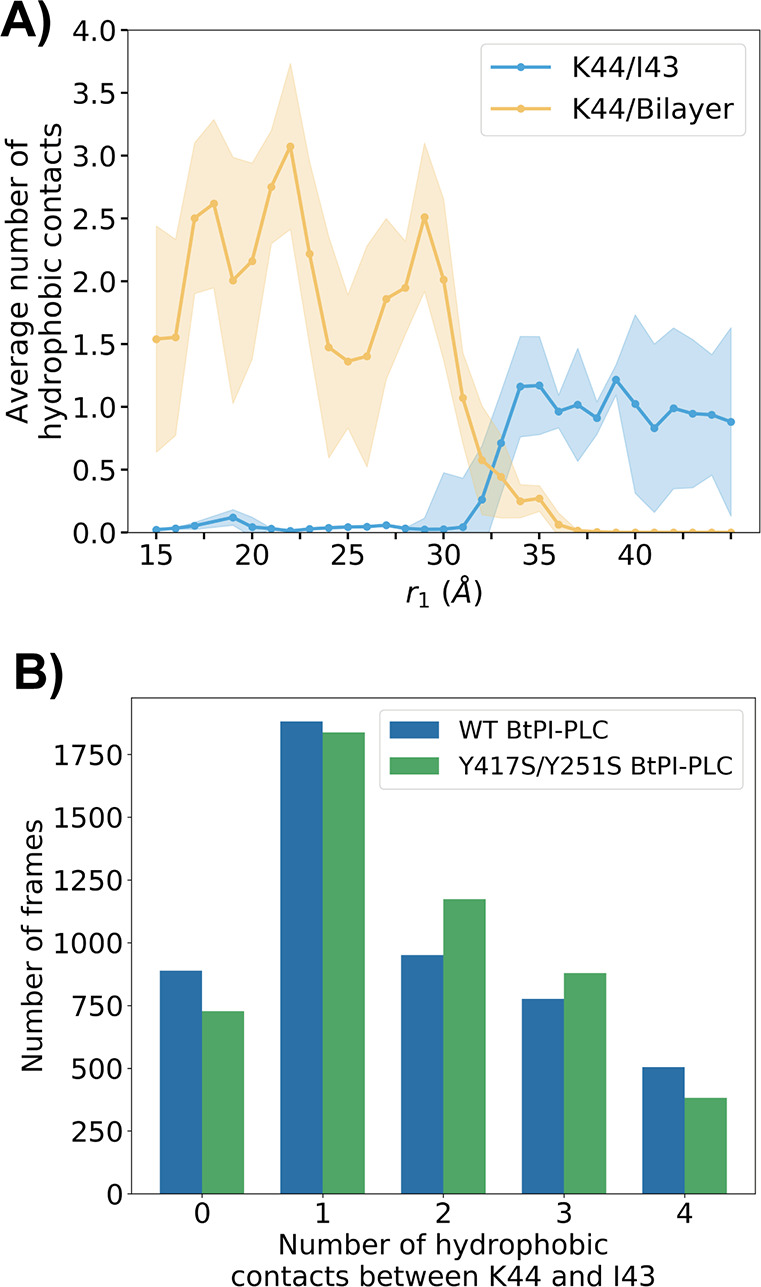
Hydrophobic contacts involving K44. (A) Average number of contacts per frame with I43 (blue) and the lipids (orange), along the separation PMF. Each point represents the average over 2000 frames collected during the separation PMF calculation. The shaded area represents the standard deviation (SD). (B) Hydrophobic contacts during equilibrium simulations of WT *Bt*PI-PLC (blue bars, system 3 in Table 1) and Y247S/Y251S *Bt*PI-PLC (green bars, system 4 in Table 1) in bulk solutions.

From *r*_1_ = 15 Å to *r*_1_ = 30 Å, the side chain of K44 engaged in hydrophobic contacts with the lipid aliphatic chains and in several hydrogen bonds (Figure 5). For values of *r*_1_ above 30 Å, the average number of hydrophobic contacts per frame between the bilayer and K44 dropped and reached 0 by *r*_1_ = 36 Å, where the protein is almost free in solution. At the same time, the average number of hydrophobic contacts per frame between K44 and I43 followed the opposite trend, from no contacts between K44 and I43 for *r*_1_ < 30 Å, where the protein is still membrane-bound, to a situation where K44 partially shields I43 from the solvent and vice versa, potentially easing the transfer of a protruding hydrophobic amino acid from membrane to water. We did observe the same contact in 50-ns long MDs simulations of the WT and Y247S/Y251S solvated in water; 80% of the frames show one or more hydrophobic contacts between R44 and I43 (Figure 5B).

**Figure 6:**
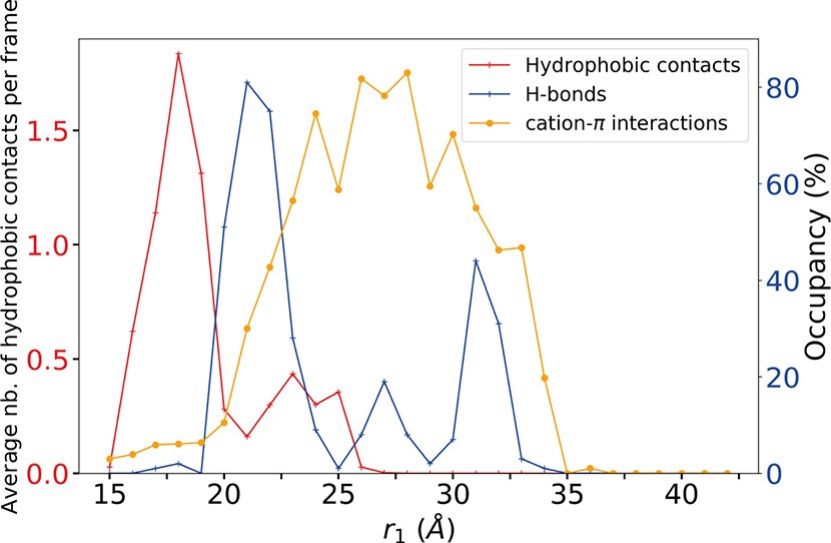
Interactions between W47 and lipids during the separation process. Occupancies are plotted for hydrogen bonds and cation−π interactions (right axis, blue labels), and the average number of contacts per frame is given for hydrophobic contacts (left axis, red labels).

**Figure 7:**
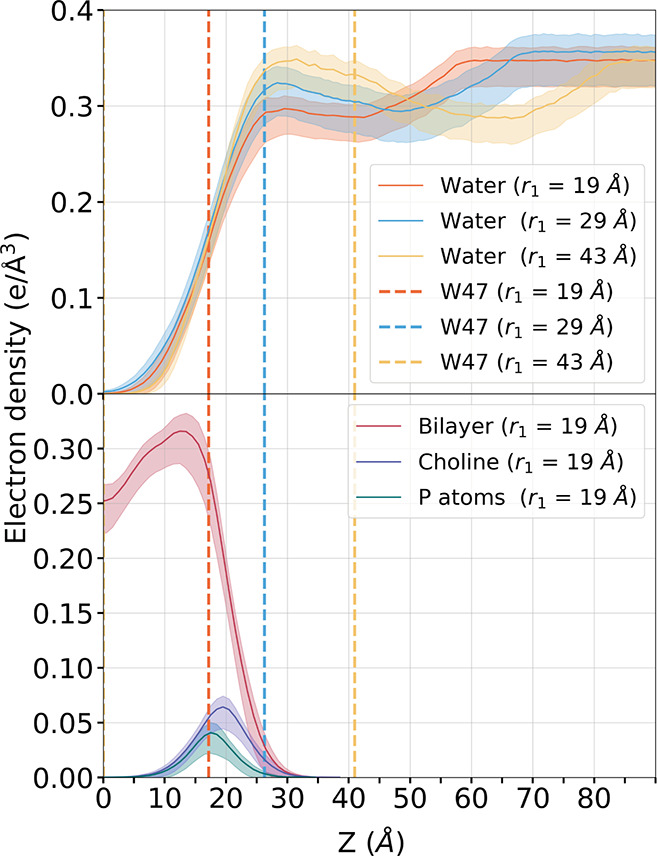
Position of W47 with respect to the bilayer and bulk along the separation PMF. The dashed lines correspond to the maximum electron density for W47 for *r*_1_ = 19, 29, or 43 Å. Electron density plots for water (top) with three different values of *r*_1_. Electron density plots for water (bottom). The electron density plots for the protein at the same step of the separation are shown in Figure S5.

We observed different types of interactions as W47 crosses the membrane interface (Figure 6). At *r*_1_ = 19 Å, when the protein was anchored in the bilayer, the W47 density peak (red dashed line) coincided with that of the phosphorus atoms (Figure 7, bottom plot, green line), and the indole group made on average more than 1.25 hydrophobic contacts with lipid chains per trajectory frame (Figure 6, red line). At *r*_1_ = 19 Å, the density of W47 also overlapped with that of the choline groups (Figure 7, bottom plot, blue line). From *r*_1_ = 21 to 22 Å, as W47 crossed the headgroup region, W47 engaged in hydrogen bonds with the phosphate groups (Figure S6 and blue line in Figure 6). These hydrogen bonds rapidly disappeared as W47 began engaging in cation−π interactions with the choline groups (Figure 6, yellow line, and Figure S6) and traversed the choline region. At *r*_1_ = 29 Å, when the density peak of W47 (Figure 7, dashed blue line) coincided with the upper limit of the choline density (blue line on bottom plot), the occupancy of cation−π adducts started decreasing (Figure 6, yellow line) until the tryptophan reached the bulk water (*r*_1_ = 34 Å) where it became completely hydrated (Figure S7).

### 3.3 DMPC Binding Sites around Y88 or Y246

**Figure 8:**
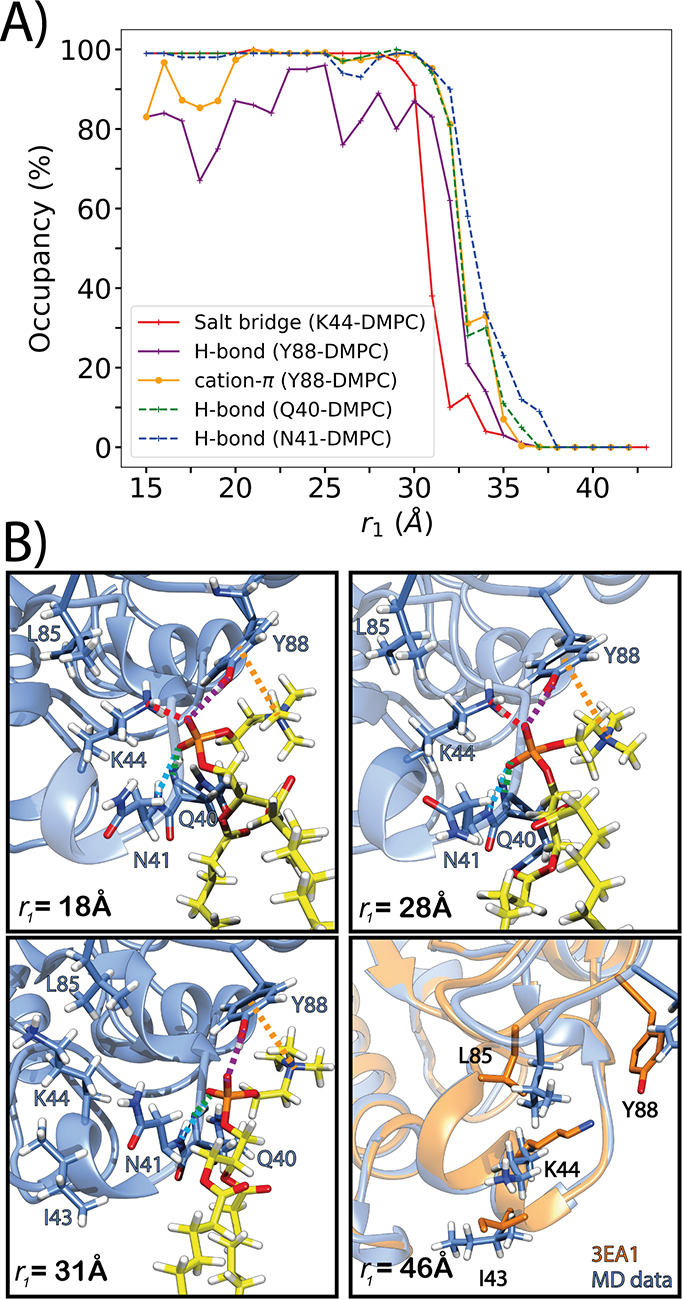
DMPC lipid interacting with Q40, N41, K44, and Y88. (A) Interactions involving backbone groups are indicated with dashed lines. All hydrogen bonds with DMPC are with the phosphate group. (B) Snapshots along the protein–membrane separation. The protein backbone is represented with a blue *cartoon*, selected side chains are shown with sticks colored by atom types (carbon atoms in blue), and sticks are used for the DMPC lipid (yellow C atoms). At *r*_1_ = 46 Å, a snapshot of the simulation (blue) is aligned with the crystal structure (PDB: 3EA1) in orange. The interactions of interest are indicated by dotted lines using the same color scheme as in panel A.

We described earlier two interaction networks, each involving one DMPC lipid that remained bound to *Bt*PI-PLC during the course of 500-ns long MDs simulations.^26^ Both interaction networks were followed during the separation PMF simulation. In the first network, the phosphate group of the PC lipid is hydrogen-bonded to Q40 and N41 through their backbone NH groups and to the (NH_3_)^+^ group of the K44 side chain via a salt bridge. Y88 establishes cation−π interactions with the choline headgroup of the same lipid, and hydrogen bonds to the phosphate with its hydroxyl group (Figure 8B). Before separation, the interaction network was stable (*r*_1_ ≤ 32–33 Å) (Figure 8A) with high occupancy for the cation−π interaction and the hydrogen bonds. The first interaction to break was the salt bridge between K44 and the phosphate group (*r*_1_ = 30 Å), followed by the Y88-DMPC hydrogen bond, the cation−π interaction, and finally the hydrogen bonds with Q40 and N41 (*r*_1_ = 35–36 Å). These observations indicate that the two hydrogen bonds plus the cation−π interaction might be necessary to maintain the lipid in place in this binding site. Also, in the second network (Figure S8), the cation−π interaction coexists with a hydrogen bond during the separation. Y246 engages in cation−π interactions with a DMPC lipid, while S244 is hydrogen-bonded to the phosphate group of that same lipid. The occupancy of the hydrogen bond decreased rapidly but was progressively replaced from *r*_1_ ≈ 30 Å by a hydrogen bond between the DMPC phosphate and the hydroxyl group of Y246 (Figure S8A). The cation−π interaction remained stable until *r*_1_ = 35 Å (occupancy ≈ 80%) except for a drop (occupancy ≈60%) coinciding with the loss of the S244 interaction.

## 4 Discussion

### 4.1 Absolute Membrane Binding Free Energy

We have calculated the absolute membrane binding free energy of *Bt*PI-PLC on a pure DMPC bilayer. This, in itself, was a challenge considering the large size of the PMP-bilayer system and the difficulties inherent to sampling the full range of the solid angle accessible to *Bt*PI-PLC during the membrane separation. We applied a framework in which *Bt*PI-PLC was progressively restrained in its bound configuration (conformation, orientation, and position) before the separation. The binding free energy was obtained within 2.6 μs of simulation, which is reasonable given the size of the system (∼140 000 atoms, system 3). In future work, the performance of the calculation may be enhanced by the use of other flavors of ABF such as the well-tempered variant of meta-eABF.^67^ The binding free energy obtained, −8.2 ± 1.4 kcal/mol, is in good agreement with earlier determined experimental values (−6.6 ± 0.2 kcal/mol) although slightly overestimated in absolute value. Even if we find this difference between computed and experimental values to be small given the calculated uncertainty, it is interesting to reflect on what its origin could be.

Fluorescence correlation spectroscopy (FCS) was used to determine equilibrium dissociation constants between *Bt*PI-PLC and small unilamellar POPC vesicles, so there are significant differences between the experimental setup and the computational setup. In order to compare our results with earlier computational studies of *Bt*PI-PLC membrane binding,^25,26^ we used a DMPC bilayer instead of POPC lipids as in the FCS experiments. Although there is no experimental data about the effect of lipid chain saturation on *Bt*PI-PLC binding to SUVs, Yang et al. showed that packing defects resulting from the introduction of conical lipids in SUVs increased the affinity of *Bt*PI-PLC for PC:PG bilayers.^68^ Earlier work using a filtration binding assay at 22 °C obtained a *K*_D_ value for *Bt*PI-PLC binding to DMPC SUVs that was 60% of the *K*_D_ for binding to POPC SUVs indicating higher affinity for DMPC SUVs.^33^ The temperature of that experiment, 22 °C, is around the *T*_m_ for the DMPC in small vesicles. The occurrence of clusters of gel-like or fluid DMPC would significantly increase the likelihood of defects and thus of PI-PLC binding. The assumption that the *K*_D_ for DMPC is 60% that of the *K*_D_ for POPC adds at most −0.3 kcal/mol to the experimental Δ*G*^0^_bind_. Clearly our computationally determined Δ*G*^0^_bind_ overestimates the binding free energy (in terms of total phospholipid) by at least 1 kcal/mol. Predicting *K*_D_ from the computational Δ*G*_bind_^0^, the accessible phospholipid *K*_D_ is 1.1 μM, leading to a *K*_D_ of 2.2 μM total phospholipid (cf. SI, part I). These values are about 10-fold lower than the lowest *K*_D_ values for pure POPC bilayers determined using FCS, our most sensitive experimental method. Similarly, since PI-PLC has a higher affinity for small, highly curved vesicles,^33^ the experimental *K*_D_ should be lower for the curved SUVs compared relative to the flat surface in the simulations, but the opposite occurs. For these reasons, it is unlikely that the differences between the experimental and computational model membranes are the cause of the difference between experimental and computed binding free energies.

Another source of error might be the molecular mechanics force field used for the simulations. The description of the electrostatic properties of lipid bilayers is known to be of limited accuracy, and in particular, the dipole potential at the membrane interface is poorly modeled by partial point charges.^69−71^ The dipole potential, which is overall positive, results from the organization of the lipid headgroups and the organization of the water molecules at the membrane surface. CHARMM36m, as other atomistic force fields for lipids,^69−71^ overestimates the dipole potential at the center of the bilayer. In the absence of experimental data, the reliability of the profile at the interface is less clear, but it has been shown that it is lower for CHARMM36m than for the polarized Drude force field. The binding of *Bt*PI-PLC to pure PC vesicles is thought to be driven mostly by the hydrophobic effect,^8^ and we earlier measured a positive electrostatic surface potential around helix αB at the IBS,^25^ which leads us to expect that the dipole potential would unfavorably contribute to the binding of *Bt*PI-PLC to PC bilayers. In that case, the slight overestimation of the computed Δ*G*_bind_^0^ might be an indication that the force resulting from the dipole potential in the phosphate region (where helix B is inserted) is underestimated.

We are confident that the CHARMM-WYF parameters for the treatment of interactions between choline headgroups and aromatic residues (F, Y, and W)^51,52^ are unlikely to contribute to an overestimation of those interactions given the earlier validation of these parameters against experimental^13,72^ and quantum mechanics data.^51,52^ On the other hand, the contribution of the inserted tryptophans W47 and W242 could be overestimated. Using FEP calculations, we indeed found that the calculated cost of replacing W242 by an alanine was overestimated (3.63 kcal/mol) compared to the experimentally derived value (2.9 ± 0.3 kcal/mol).^13^

### 4.2 Protein–Lipid Interactions

One major advantage of using a geometrical route and an atomistic force field to compute the free energy of binding is the insight we gain into PMP-membrane interactions at the atomic level of details. While we cannot completely rule out an effect of the constraints on the desorption mechanism, the fact that *Bt*PI-PLC does not undergo large conformational changes upon binding, the qualitative agreement between our proposed mechanism and earlier experimental and computational data indicates that the effect of the constraints could be minimal.

The role of lysines at interfacial binding sites in PMPs has long been thought to be primarily an electrostatic one; being part of a cluster of basic amino acids, they contribute to nonspecific electrostatic interactions driving the protein to orient in an insertion-competent orientation. In this mechanism, the contribution of lysines to the membrane affinity has been estimated as ca. 1 kcal/mol.^73^ In *Bt*PI-PLC, we showed earlier that the mutation of K44 to alanine caused an ca. 55-fold decrease of the protein affinity for PC:PG 80:20 liposomes, corresponding to a contribution to the affinity of around 2.4 kcal/mol.^25^ For vesicles composed only of PC lipids, the K44A mutation caused a 17-fold decrease of the protein affinity, or ca. 1.6 kcal/mol.^25^ Those numbers cannot be solely explained by electrostatic interactions with the lipids. We suggested for lysines a role that goes beyond the traditional nonspecific electrostatic contributions to peripheral membrane binding and instead takes advantage of their partly aliphatic side chain as hydrophobic anchors. This hydrophobic character of the K44 side chain is underlined by its behavior in the separation simulation: the unwinding of the interactions of K44 with the lipids exposed its side chain which immediately engaged in hydrophobic contacts with the side chain of I43, to prevent an unfavorable exposure to a solvent.

The strongest cation−π interactions between *Bt*PI-PLC tyrosines and choline lipids have an estimated contribution of 3.1 and 2.7 kcal/mol for Y88 and Y246, respectively.^26^ The DMPC lipids involved in cation−π interactions with Y88 and Y246 also tightly interacted with other amino acids through hydrogen bonds. These interactions were among the last to unravel during the protein–membrane separation. They could also be accompanied by a hydrogen bond between the tyrosine hydroxyl group and the phosphate. The concomitance of these interactions induced a strong and specific interaction between the PC headgroup and the tyrosines. The interaction network around Y88 appears to be a lipid-binding site rather than the result of opportunistic interactions with whichever lipid is available in the vicinity. For Y246, the interaction involved fewer amino acids, but as for Y88, the same lipid occupied the site from the beginning to the end of the separation PMF simulation. Before we recognized the importance of tyrosine for PC recognition, we reported that intramolecular cross-linking of the protein in the presence of diheptanoylphosphatidylcholine micelles trapped two of the short-chain PC molecules on the protein.^30^ Our evolving understanding of the lipid-specificity of *Bt*PI-PLC based on past studies and the present results leads us to propose that the two tyrosines Y88 and Y246 play key roles in these binding sites.

From PMFs of amino acid side chain analogs across lipid bilayers,^45^ we know that tyrosine and tryptophan have a deep global minimum below the phosphate groups, more precisely in the region containing the carbonyl groups and the initial part of the polar headgroup density. For both amino acids, the free energy of transfer is higher in the region of the phosphates and cholines (by 4.5 and 3.3 kcal/mol for Trp and Tyr, respectively). In that region, tyrosine and tryptophan from peripheral proteins can engage in cation−π interactions with choline groups.^13^ We showed that when they engage in cation−π interactions their contribution to the protein–membrane affinity is at least comparable to their contribution when they insert under the phosphate groups. In this work, we observed that W47 engaged in different types of interactions depending on the interface regions it finds itself in, taking advantage of its ability to engage in hydrophobic contacts below the phosphates, and then hydrogen bonds with phosphates and cation−π interactions with choline groups. These interactions were interrupted when W47 was in bulk water and surrounded by explicit water molecules. Based on the behavior of W47 during separation of the protein from the bilayer, we propose that this might be a strategy facilitating tryptophan adsorption onto bilayers and that indole rings engaging in cation−π interactions high up at the interface might be a means to facilitate their path through the interfacial region on their way to deeper insertion in the carbonyl region.

In general, earlier studies have shown that binding is significantly impaired if helix B does not contain at least one exposed aromatic amino acid (W47) and one exposed hydrophobe.^34^ The latter is I43, but it could be substituted by a tryptophan (I43W) without loss of affinity,^34,74^ and likewise W47 could be substituted by a phenylalanine (W47F).^75^ However, substituting W47 by an isoleucine (W47I) increased the *K*_D_ by a factor of 3,^75^ unless I43 was also substituted by a tryptophan (double mutant I43I/W47I).^34^ The necessity for aromatic amino acids could be explained by their ability to engage in interactions with choline headgroups.

## 5 Conclusion

In this contribution, we determined for the first time, to our knowledge, the absolute membrane binding free energy Δ*G*_bind_^0^ of a PMP (*Bt*PI-PLC) partitioning onto a phospholipid bilayer, using an atomistic force field and the so-called geometrical route.^10^ The computed Δ*G*_bind_^0^ is in good agreement with the available experimental data. Our study thus demonstrates the potential of our computational framework for the estimation of PMP-membrane affinities in a case where the PMP IBS is unique and well-defined, and the PMP does not undergo significant conformational changes upon binding to the membrane. Using this framework to investigate and compare several prospective IBSs is in principle possible, though potentially computationally demanding. The high computational cost of using an atomistic force field is balanced by the opportunity to gain new insights into the mechanism whereby protein–lipid interactions break down as the protein desorbs from the bilayer. We showed how hydrophobes engage in an array of interactions to ease their passage through the polar headgroup region: a tryptophan engaged in transient cation−π interactions with a lipid choline group, and an isoleucine side chain sought contact with the aliphatic chain of a neighboring lysine. This work also underscores the importance of atomistic force fields to enhance our understanding of lipid recognition by PMPs.

## 6 Data and Software Availability

All data and software used in this study are available freely. Data sources and identifiers are given in the text.

## Supporting Information Available

Details of calculation of experimental *ΔG*_bind_^0^ for *Bt*PI-PLC (PDF), calculation of computationally determined *ΔG*_bind_^0^, and additional analyses of molecular dynamics trajectories and of X-ray structures (side chain orientations in multiple *Bt*PI-PLC structures) (PDF)
