# Supplementary material for: Standard Binding Free Energy and Membrane Desorption Mechanism for a Phospholipase C
Source: J Chem Inf Model. 2022 Mar 28;62(24):6602–13. doi: 10.1021/acs.jcim.1c01543 (PMC9795555; doi:10.1021/acs.jcim.1c01543)
Supplement: Supplementary file 1 — ci1c01543_si_001.pdf [file ci1c01543_si_001.pdf]

**Supporting Information**

# Standard binding free energy and membrane desorption mechanism for a phospholipase C

*Emmanuel E. Moutoussamy[1,2], Hanif M. Khan[†1,2], Mary F. Roberts[3], Anne Gershenson[#4], Christophe Chipot[5,6], Nathalie Reuter[*2,7]*

[1] Department of Biological Sciences, University of Bergen, N-5020 Bergen, Norway

[2] Computational Biology Unit, Department of Informatics, University of Bergen, N-5020 Bergen, Norway

[3] Department of Chemistry, Boston College, Chestnut Hill, Massachusetts 02467, United States

[4] Department of Biochemistry and Molecular Biology, University of Massachusetts Amherst, Amherst, Massachusetts 01003, United States

[5] Laboratoire International Associé Centre National de la Recherche Scientifique et University of Illinois at Urbana-Champaign, Unité Mixte de Recherche n 7019, Université de Lorraine, BP 70239, 54506 Vandœuvre-lès-Nancy cedex, France

[6] Department of Physics, University of Illinois, Urbana, Illinois, United States

[7] Department of Chemistry, University of Bergen, N-5020 Bergen, Norway

PRESENT ADRESSES

† Hanif Muhammad Khan - Centre for Molecular Simulation, Department of Biological Sciences, University of Calgary, Calgary, Canada

# Anne Gershenson - NIGMS/NIH Bethesda, MD 20892 USA

## *I. Calculation of the experimental $\Delta G^{o}_{bind}$ for BtPI-PLC binding to POPC SUVs*

**Table S1.** Dissociation constants ($K_D$) of *Bt*PI-PLC on pure PC vesicles.

| Reference | $K_D$ ($\mu M$) | $\Delta G^{0}_{bind}$ (kcal/mol)[a] |
|---|---|---|
| Cheng *et al.* (2013) [1] | 25 | -6.6 |
| Grauffel *et al.* (2013) [2] | 16±3 | -6.8 |
| He *et al.* (2015) [3] | 30±6 | -6.5 |
| Khan *et al.* (2016) [4] | 26±5 | -6.6 |
| Average: | 24 ± 6 | -6.6 ± 0.2 |

[a] Average $\Delta G^{0}_{bind}$ from assuming that SUVs have 0.75 or 0.65 of the POPC in the outer leaflet.

Although the MD simulations were performed for the enzyme binding to a DMPC bilayer, most of our FCS data has used vesicles containing POPC (with WT *Bt*PI-PLC binding to pure POPC SUVs carried out as a control any time a mutant protein was examined). An earlier binding study using a filtration assay at 22°C showed that the $K_{D,app}$ for *Bt*PI-PLC binding to DMPC SUVs was similar to that for POPC SUVs measured in the same conditions[5]. The average $K_D$ calculated from all of our published FCS experiments of *Bt*PI-PLC binding to pure POPC SUVs is 24±6 µM (Table S1). That value is in terms of the total phospholipid concentration. These small sonicated vesicles are not homogeneous, but have a range of sizes with > 80% of the vesicle diameters between 150 and 300 nm[6]. For typical POPC SUV preparations, $^{31}P$ NMR analysis of POPC SUVs with $Pr^{3+}$ added can separate POPC resonances for the inner and outer monolayers. For these sonicated vesicles, those analyses are consistent with between 0.65 and 0.75 of the total lipid being in the outer monolayer. Since only that fraction of POPC is accessible to the *Bt*PI-PLC, the average $K_D$ ranges from 15.6 µM to 18 µM. This corrected $K_D$ was then used to estimate the molar partition coefficient for *Bt*PI-PLC binding to the vesicles $K_p$ as follows[7].

$$K_p = [K_D \text{ x } N_A \text{ x } v_{lipid}]^{-1}$$

where $v_{lipid}$ is the volume of one lipid molecule, 1256 Å$^3$ for POPC[7], and $N_A$ is Avogadro's number. For *Bt*PI-PLC binding to POPC SUVs, the average $K_p$ is then between 76,800 and 88,600 leading to an average $\Delta G^{o}_{bind}$ of -6.6 kcal/mol.

*II. Absolute binding free energy calculation*

- Explicit derivation of the binding free energy from PMFs contributions from adding restraints in the bound state:

$$e^{\beta G_c^{site}} = \frac{\int_{\text{site}} \text{d1} \int \text{dX} e^{-\beta U}}{\int_{\text{site}} \text{d1} \int \text{dX} e^{-\beta[U + u_c]}} = \frac{0.13999}{0.0009690} = 144.380 \rightarrow \boldsymbol{G_c^{site} = 2.9643\ kcal/mol}$$

$$e^{\beta G_{\Theta_1}^{site}} = \frac{\int_{\text{site}} \text{d1} \int \text{dX} e^{-\beta[U + u_c]}}{\int_{\text{site}} \text{d1} \int \text{dX} e^{-\beta[U + u_c + u_{\Theta_1}]}} = \frac{2.178584}{2.039155} = 1.0683 \rightarrow \boldsymbol{G_{\Theta_1}^{site} = 0.0394\ kcal/mol}$$

$$e^{\beta G_{\Phi_1}^{site}} = \frac{\int_{\text{site}} \text{d1} \int \text{dX} e^{-\beta[U + u_c + u_{\Theta 1}]}}{\int_{\text{site}} \text{d1} \int \text{dX} e^{-\beta[U + u_c + u_{\Theta 1} + u_{\Phi_1}]}} = \frac{2.508965}{2.288154} = 1.0965 \rightarrow \boldsymbol{G_{\Phi_1}^{site} = 0.05492\ kcal/mol}$$

$$e^{\beta G_{\Psi_1}^{site}} = \frac{\int_{\text{site}} \text{d1} \int \text{dX} e^{-\beta[U + u_c + u_{\Theta_1} + u_{\Phi_1}]}}{\int_{\text{site}} \text{d1} \int \text{dX} e^{-\beta[U + u_c + u_{\Theta_1} + u_{\Phi 1} + u_{\Psi_1}]}} = \frac{3.82029}{2.90389} = 1.31557 \rightarrow \boldsymbol{G_{\Psi_1}^{site} = 0.1635\ kcal/mol}$$

$$e^{\beta G_{\theta_1}^{site}} = \frac{\int_{\text{site}} \text{d1} \int \text{dX} e^{-\beta[U + u_c + u_o]}}{\int_{\text{site}} \text{d1} \int \text{dX} e^{-\beta[U + u_c + u_o + u_{\theta_1}]}} = \frac{1.88267}{1.77144} = 1.06279 \rightarrow \boldsymbol{G_{\theta_1}^{site} = 0.0363\ kcal/mol}$$

$$e^{\beta G_{\varphi_1}^{site}} = \frac{\int_{\text{site}} \text{d1} \int \text{dX} e^{-\beta[U + u_c + u_o + u_{\theta_1}]}}{\int_{\text{site}} \text{d1} \int \text{dX} e^{-\beta[U + u_c + u_o + u_{\theta_1} + u_{\varphi_1}]}} = \frac{2.94475}{2.68063} = 1.09852 \rightarrow \boldsymbol{G_{\varphi_1}^{site} = 0.0560\ kcal/mol}$$

- Separation PMF:

$$\frac{\int_{\text{site}} \text{d1} \int \text{dX} e^{-\beta[U + u_c + u_o + u_a]}}{\int_{\text{bulk}} \text{d1} \delta(r_1 - r_1^*) \int dX e^{-\beta[U + u_c + u_o]}} = S^* I^*$$

- I* and S* terms with three different values of $r_1$* (41, 44 and 47 Å):

$$I^*\ (r_1{}^* = 41\text{Å}) = \int \text{d}r e^{-\beta[W(r_1) - W(r_1{}^*)]} = \int \text{d}r e^{-\beta[W(r_1) - W(41.0)]} = \int \text{d}r e^{-\beta[W(r_1) - 21.63]} = \mathbf{3.75126 \times 10^{14}}\ \text{Å}$$

$$I^* \ (r_1{}^* = 44\text{Å}) = \int \text{dr}e^{-\beta[W(r_1) - W(r_1{}^*)]}$$

$$= \int \text{dr}e^{-\beta[W(r_1) - W(41.0)]} = \int dre^{-\beta[W(r_1) - 21.63]} = \mathbf{5.9902} \times \mathbf{10^{14}} \text{ Å}$$

$$I^* \ (r_1{}^* = 46\text{Å}) = \int dre^{-\beta[W(r_1) - W(r_1{}^*)]}$$

$$= \int dre^{-\beta[W(r_1) - W(41.0)]} = \int dre^{-\beta[W(r_1) - 21.63]} = \mathbf{3.37814} \times \mathbf{10^{14}} \text{ Å}$$

$$S^*(r_1{}^*$$

$$= 41 \text{ Å}) = (r_1{}^*)^2 \int_0^{\frac{\boldsymbol{\pi}}{\mathbf{2}}} \sin{(\theta_1)} d\theta_1 \int_0^{2\pi} d\varphi_1 e^{-\beta u_a(\theta_1,\varphi_1)} = (r_1{}^*)^2 \int_0^{\frac{\pi}{2}} \sin d\theta_1$$

$$e^{-\beta\left(0.5 \times 0.1 \times \left(\frac{180}{\pi}\right)^2\right) \times \left(\theta_1 - 0.0785 \times \frac{\pi}{180}\right)^2} \times \int_0^{2\pi} d\varphi_1 e$$

$$-\beta \times \left(0.5 * 0.1 * \left(\frac{180}{\pi}\right)^2\right) \times \left(\varphi_1 - 5.427 \times \frac{\pi}{180}\right)^2$$

$$= (41.0)^2 \times 0.008431586 \times 0.1068191 = \mathbf{1.84016} \text{ Å}^{\mathbf{2}}$$

$$S^*(r_1{}^* = 44 \text{ Å}) = (44.0)^2 \times 0.008431586 \times 0.1068191 = \mathbf{2.11930} \text{ Å}^{\mathbf{2}}$$
$$S^*(r_1{}^* = 46 \text{ Å}) = (46.0)^2 \times 0.008431586 \times 0.1068191 = \mathbf{2.31634} \text{ Å}^{\mathbf{2}}$$

$$S^*I^*(r_1{}^* = 41 \text{ Å}) = (1.84016) \times (3.75126 \times 10^{14}) = \mathbf{6.902940} \times \mathbf{10^{14}} \text{ Å}^{\mathbf{3}}$$
$$S^*I^*(r_1{}^* = 44 \text{ Å}) = (2.11930\ ) \times (5.9902 \times 10^{14}) = \mathbf{1.269525} \times \mathbf{10^{15}} \text{ Å}^{\mathbf{3}}$$
$$S^*I^*(r_1{}^* = 46 \text{ Å}) = (2.31634) \times (3.37814 \times 10^{14}) = \mathbf{7.824967} \times \mathbf{10^{14}} \text{ Å}^{\mathbf{3}}$$

- Contributions from removing restraints in the unbound state:

$$e^{-\beta G_o^{bulk}} = \frac{1}{8\pi^2}\int_0^{\pi}\sin(\Theta_1)d\Theta_1 \int_0^{2\pi} d\Theta_1 \int_0^{2\pi} d\Psi_1\, e^{-\beta u_o(\Theta_1,\Phi_1,\Psi_1)} = \frac{1}{8\pi^2}\int_0^{\pi}\sin(\Theta_1)d\Theta_1\, e^{-\beta\times\left(0.5\times 0.1\times\left(\frac{180}{\pi}\right)^2\right)\times\left(\Theta_1-10\times\left(\frac{\pi}{180}\right)\right)^2} \times \int_0^{2\pi} d\Phi_1 e^{-\beta\times\left(0.5\times 0.1\times\left(\frac{180}{\pi}\right)^2\right)\times\left(\Phi_1-12.5\times\left(\frac{\pi}{180}\right)\right)^2} \times \int_0^{2\pi} d\Psi_1 e^{-\beta\times\left(0.5\times 0.1\times\left(\frac{180}{\pi}\right)^2\right)\times\left(\Psi_1-8.5\times\left(\frac{\pi}{180}\right)\right)^2} = \frac{1}{8\pi^2}(0.0185)\times(0.1068)\times(0.1067) = 2.67747\times 10^6 \rightarrow G_{bulk}^{o} = \mathbf{7.649\ kcal/mol}$$

$$e^{-\beta G_c^{bulk}} = \frac{\int_{\text{bulk}} d1\delta(\text{r}_1-\text{r}_1^*)\int \text{dX}e^{-\beta[U+u_c]}}{\int_{\text{bulk}} d1\delta(\text{r}_1-\text{r}_1^*)\int \text{dX}e^{-\beta U}} = \frac{0.23852}{4.73672\times 10^{-4}} = 503.567 \rightarrow \text{G}_\text{c}^{bulk} = \mathbf{3.7091\ kcal/mol}$$

- Final calculation of the binding constant and free energy

$$K_{\text{eq}}(r_1{}^* = 41\ \text{Å}) = S^* I^* e^{-\beta[G_c^{\text{bulk}}+G_o^{\text{bulk}}-G_a^{\text{site}}-G_o^{\text{site}}-G_c^{\text{site}}]} = S^* I^* \times e^{-\beta[3.7091\ +\ 7.649\ -\ (0.0363\ +\ 0.0560)\ -\ (0.0394\ +0.05492+0.1635)\ -\ 2.9643]} = S^* I^* \times (1.38128\times 10^{-6}) = (6.902940\times 10^{14})\times(1.38128\times 10^{-6}) = \mathbf{9.5349\times 10^{8}}\ \text{Å}^{\mathbf{3}}$$

$$K_{\text{eq}}(r_1{}^* = 44\ \text{Å}) = (1.269525\times 10^{15})\times(1.38128\times 10^{-6}) = \mathbf{1.7535\times 10^{9}}\ \text{Å}^{\mathbf{3}}$$

$$K_{\text{eq}}(r_1{}^* = 46\ \text{Å}) = (7.824967\times 10^{14})\times(1.38128\times 10^{-6}) = \mathbf{1.0808\times 10^{9}}\ \text{Å}^{\mathbf{3}}$$

$$\Delta \boldsymbol{G}_{\textbf{bind}}^{\mathbf{0}}(r_1{}^* = \mathbf{41}\ \text{Å}) = -\mathbf{8.168}\ \boldsymbol{kcal/mol}$$
$$\Delta \boldsymbol{G}_{\textbf{bind}}^{\mathbf{0}}(r_1{}^* = \mathbf{44}\ \text{Å}) = -\mathbf{8.544}\ \boldsymbol{kcal/mol}$$
$$\Delta \boldsymbol{G}_{\textbf{bind}}^{\mathbf{0}}(r_1{}^* = \mathbf{46}\ \text{Å}) = -\mathbf{8.246}\ \boldsymbol{kcal/mol}$$

*III. Analysis of MD trajectories and X-ray structures*

**Figure S1.** Backbone distance RMSD with respect to the last structure after the second equilibration step for WT *Bt*PI-PLC in water (system 3) and Y247S/Y251S *Bt*PI-PLC (system 4).

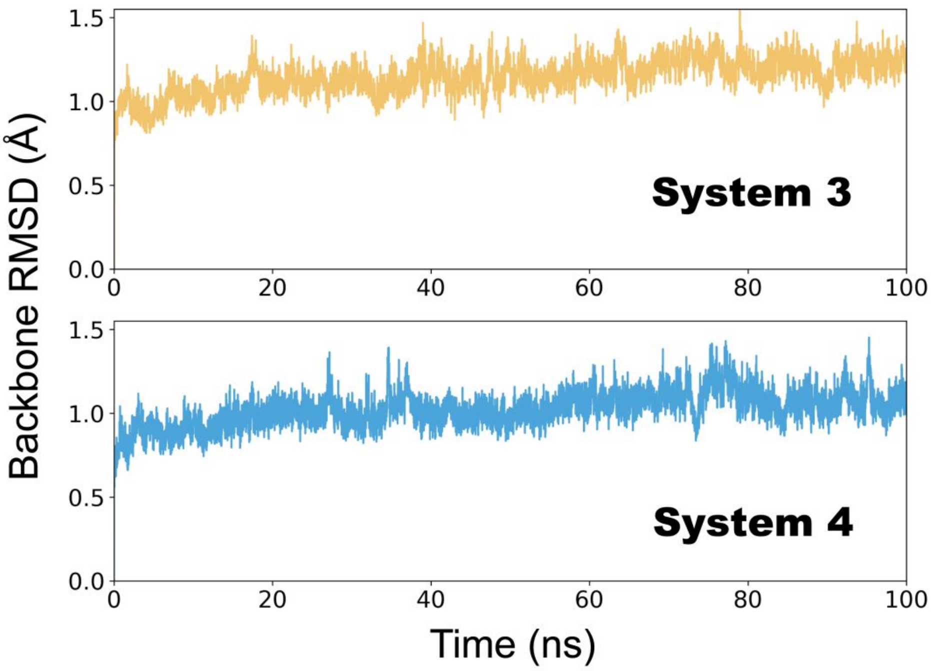


**Figure S2.** Distance between protein center of mass (COM) and the COM of the upper phosphate plane during the equilibration of the *large Bt*PI-PLC-bilayer system (system 2). The dashed line represents the average distance.

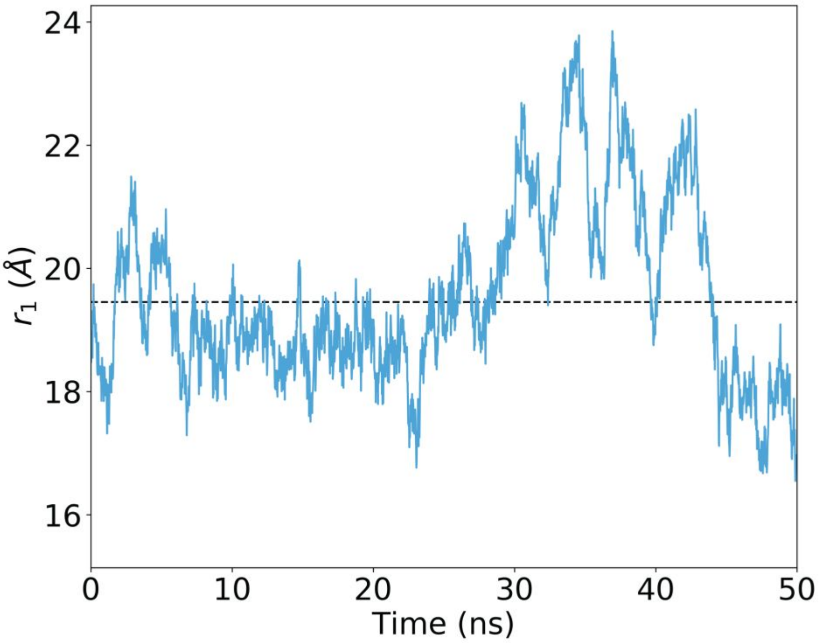

**Figure S3.** Correspondence between $r_1$ and the minimum atomic distance between the protein and the bilayer. $r_1$ is the distance between the protein center of mass (COM) and the COM of the upper phosphate plane.

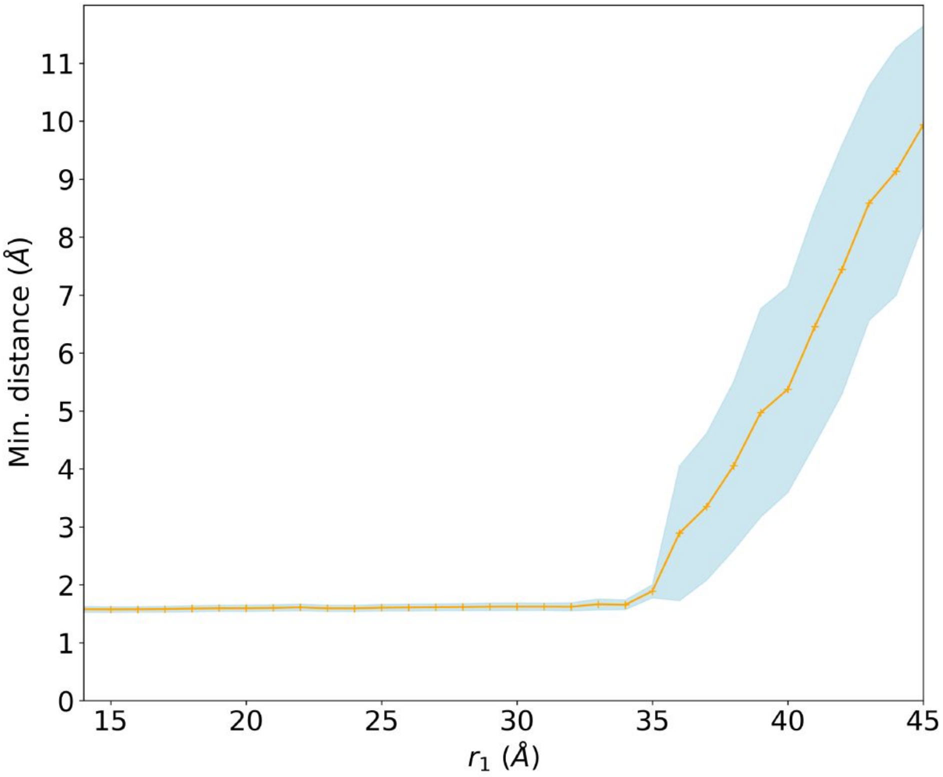


**Figure S4.** Side chain orientation for I43, K44, L85 and Y88 in X-ray structures of *Bt*PI-PLC variants. The PDB IDs are 1T6M (yellow, R70D), 3EA1 (purple, Y247S/Y251S), 3EA2 (red, Y247S/Y251S) and 3EA3 (green, Y246S/Y247S/Y248S/Y251S).

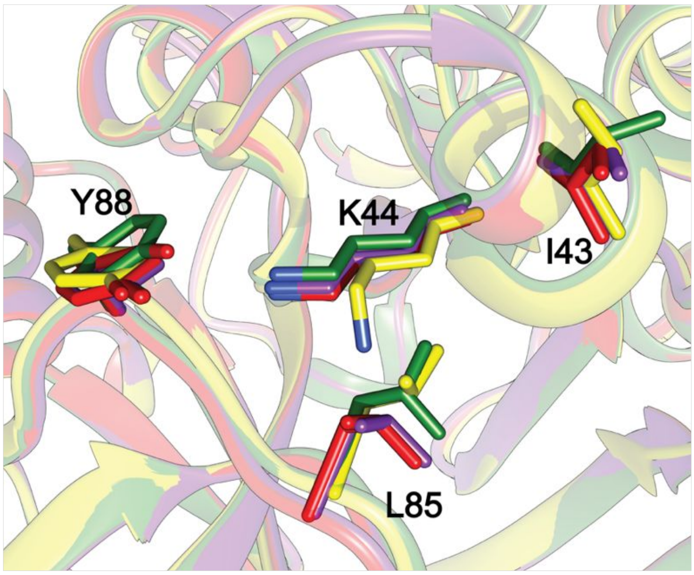

**Table S2.** Occupancies of DMPC-tyrosine cation-π interactions during the separation PMF at r = 18 Å, and in equilibrium simulations reported earlier [4] (Khan *et al.*, 2016).

| Residue | Occupancy (%) Khan *et al.*, 2016 [4] | Occupancy (%) Separation PMF at r = 18Å |
|---|---|---|
| Y86 | 11.9 | 14.7 |
| Y88 | 93.1 | 85.3 |
| Y118 | 12.9 | 8.5 |
| Y200 | 43.0 | 34.9 |
| Y204 | 22.3 | 26.7 |
| Y246 | 80.9 | 77.9 |
| Y247 | 12.7 | 7.6 |
| Y251 | 47.0 | 60.6 |

**Figure S5.** Electron density plots for protein and bilayer along the separation PMF: the density of the bilayer is shown with solid lines and the density of *Bt*PI-PLC with dashed lines, at $r_1$ = 19, 29 and 43 Å in blue, yellow and red, respectively.

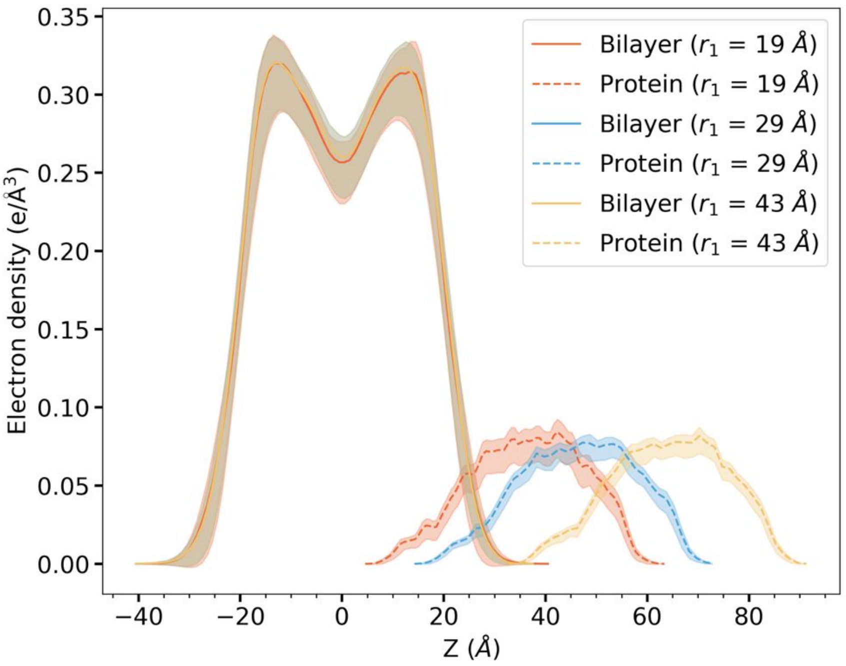

**Figure S6** Hydrogen bond (left) and cation-π interaction (right) between DMPC lipids and W47 along the protein-membrane separation. The protein is represented in blue and the PC lipid in yellow.

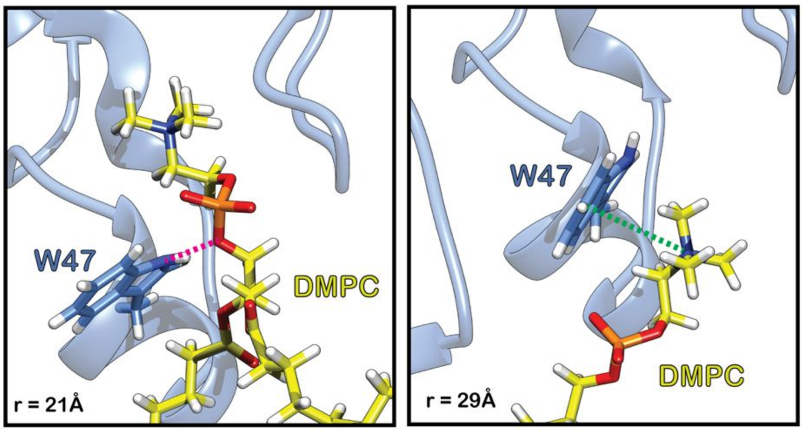


**Figure S7. Average number of water molecules around W47 along the separation process**. (A) Average number of water molecules per frame and (B) snapshot at $r_1$ = 29 Å and (C) $r_1$ = 43 Å. The water molecules are represented in green, W47 in blue and the interacting DMPC lipid in yellow.

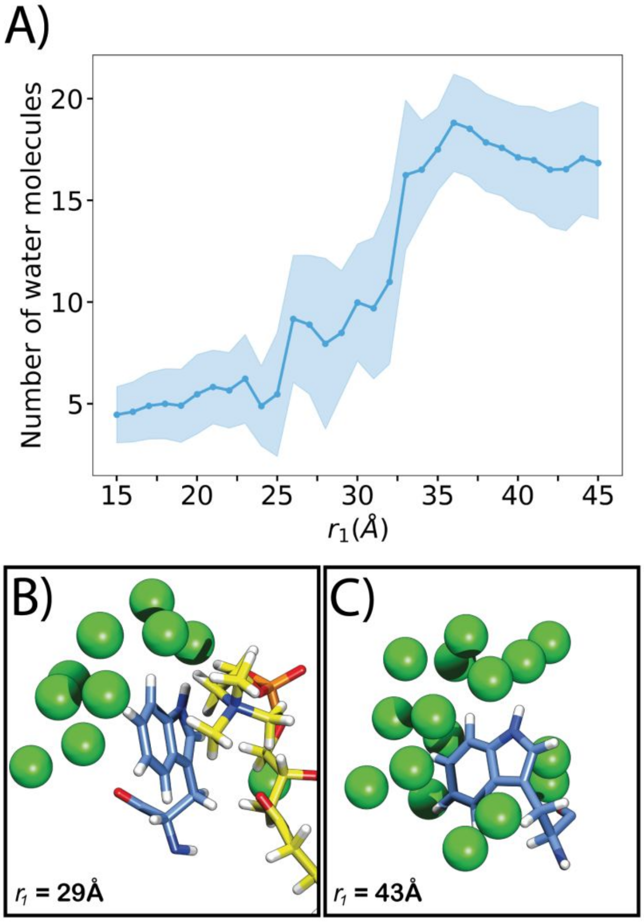

**Figure S8. DMPC lipid interacting with S244 and Y246.** (A) Changes along the separation PMF. $r_1$ is the distance between the protein COM and the average upper phosphate plane. The hydrogen bond is with the DMPC phosphate group. (B) Snapshots along the protein-membrane separation. The protein backbone is represented with blue *cartoon*, sticks colored by atom types are shown for selected side chains (carbon atoms in blue) and a PC lipid (yellow C atoms). At $r_1 = 46$ Å, a snapshot of the simulation (blue) is aligned with the crystal structure (PDB: 3EA1) in orange. The hydrogen bonds and cation-π interactions are indicated by dotted lines using the same color scheme as in panel A.

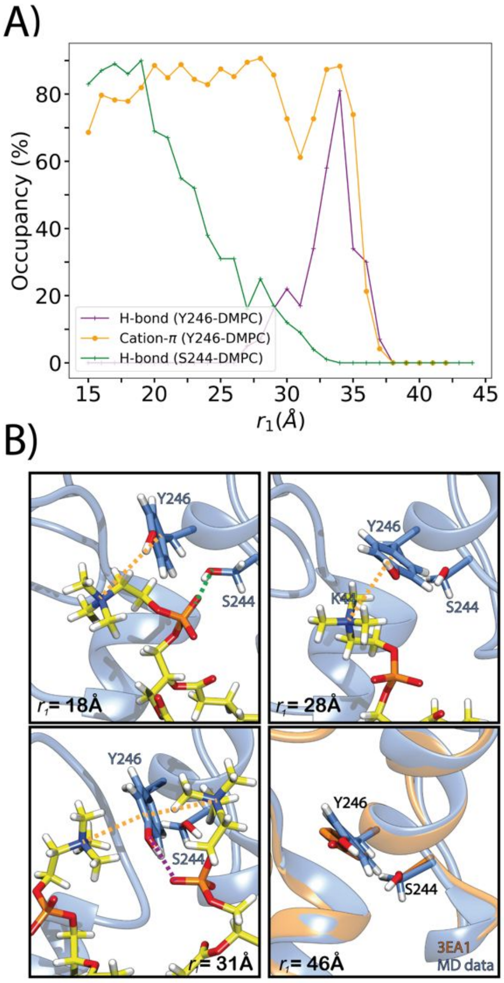
A)
Occupancy (%)
H-bond (Y246-DMPC)
Cation-π (Y246-DMPC)
H-bond (S244-DMPC)
r1(Å)
B)
Y246
S244
r1= 18Å
Y246
S244
r1= 28Å
Y246
S244
r1= 31Å
Y246
S244
3EA1
MD data
r1= 46Å

**References**


(1) Cheng, J.; Karri, S.; Grauffel, C.; Wang, F.; Reuter, N.; Roberts, M. F.; Wintrode, P. L.; Gershenson, A. Does Changing the Predicted Dynamics of a Phospholipase C Alter Activity and Membrane Binding? *Biophys. J.* **2013**, *104* (1), 185–195. https://doi.org/10.1016/j.bpj.2012.11.015.

(2) Grauffel, C.; Yang, B.; He, T.; Roberts, M. F.; Gershenson, A.; Reuter, N. Cation-π Interactions as Lipid-Specific Anchors for Phosphatidylinositol-Specific Phospholipase C. *J. Am. Chem. Soc.* **2013**, *135* (15), 5740–5750. https://doi.org/10.1021/ja312656v.

(3) He, T.; Gershenson, A.; Eyles, S. J.; Lee, Y.-J.; Liu, W. R.; Wang, J.; Gao, J.; Roberts, M. F. Fluorinated Aromatic Amino Acids Distinguish Cation-π Interactions from Membrane Insertion. *J. Biol. Chem.* **2015**, *290* (31), 19334–19342. https://doi.org/10.1074/jbc.M115.668343.

(4) Khan, H. M.; He, T.; Fuglebakk, E.; Grauffel, C.; Yang, B.; Roberts, M. F.; Gershenson, A.; Reuter, N. A Role for Weak Electrostatic Interactions in Peripheral Membrane Protein Binding. *Biophys. J.* **2016**, *110* (6), 1367–1378. https://doi.org/10.1016/j.bpj.2016.02.020.

(5) Wehbi, H.; Feng, J.; Kolbeck, J.; Ananthanarayanan, B.; Cho, W.; Roberts, M. F. Investigating the Interfacial Binding of Bacterial Phosphatidylinositol-Specific Phospholipase C. *Biochemistry* **2003**, *42* (31), 9374–9382. https://doi.org/10.1021/bi034195+.

(6) Yang, B.; Pu, M.; Khan, H. M.; Friedman, L.; Reuter, N.; Roberts, M. F.; Gershenson, A. Quantifying Transient Interactions between Bacillus Phosphatidylinositol-Specific Phospholipase-C and Phosphatidylcholine-Rich Vesicles. *J. Am. Chem. Soc.* **2015**, *137* (1), 14–17. https://doi.org/10.1021/ja508631n.

(7) Middleton, E. R.; Rhoades, E. Effects of Curvature and Composition on α-Synuclein Binding to Lipid Vesicles. *Biophys. J.* **2010**, *99* (7). https://doi.org/10.1016/j.bpj.2010.07.056.
